# Glucose Intolerance after a Recent History of Gestational Diabetes Based on the 2013 WHO Criteria

**DOI:** 10.1371/journal.pone.0157272

**Published:** 2016-06-10

**Authors:** Katrien Benhalima, Katleen Jegers, Roland Devlieger, Johan Verhaeghe, Chantal Mathieu

**Affiliations:** 1 Department of Endocrinology, UZ Gasthuisberg, KU Leuven, Leuven, Belgium; 2 Department of Obstetrics & Gynecology, UZ Gasthuisberg, KU Leuven, Leuven, Belgium; Baylor College of Medicine, UNITED STATES

## Abstract

**Aims:**

Uncertainty exists on the prevalence of glucose intolerance in women with a recent diagnosis of gestational diabetes (GDM) based on a two-step screening strategy and the 2013 World Health Organization (WHO) criteria. Our aim was to evaluate the uptake of postpartum screening, the prevalence and the risk factors for glucose intolerance in women with a recent history of GDM.

**Methods:**

Retrospective analysis of the medical records of women with a recent history of GDM diagnosed in a universal two-step screening strategy with the 2013 WHO criteria. All women with a history of GDM are advised to undergo a 75g oral glucose tolerance test (OGTT) around 12 weeks postpartum. Indices of insulin sensitivity (the Matsuda index and the reciprocal of the homeostasis model assessment of insulin resistance, 1/HOMA-IR) and an index of beta-cell function, the Insulin Secretion-Sensitivity Index-2 (ISSI-2) were calculated based on the OGTT postpartum. Multivariable logistic regression was used to adjust for confounders such as age, BMI, ethnicity and breastfeeding.

**Results:**

Of the 191 women with GDM, 29.3% (56) did not attend the scheduled postpartum OGTT. These women had a higher BMI (28.6 ±6.8 vs. 26.2 ± 5.6, p = 0.015), were more often from an ethnic minority (EM) background (41.1% vs. 25.2%, p = 0.029) and smoked more often during pregnancy (14.3% vs. 2.2%, p = 0.001) than women who attended the OGTT postpartum. Of all women (135) who received an OGTT postpartum, 42.2% (57) had prediabetes (11.9% impaired fasting glucose, 24.4% impaired glucose tolerance and 5.9% both impaired fasting and impaired glucose tolerance) and 1.5% (2) had overt diabetes. Compared to women with a normal OGTT postpartum, women with glucose intolerance were older (32.5±4.3 vs. 30.8±4.8 years, p = 0.049), were more often obese (34.5% vs. 17.3%, p = 0.023), were more often from an EM background (33.9% vs. 18.4%, p = 0.040), less often breastfed (69.5% vs. 84.2%, p = 0.041) and had more often an abnormal fasting glycaemia at the time of the OGTT in pregnancy (55.6% vs. 37.3%, p = 0.040). In the multivariable logistic regression, an EM background [OR = 2.76 (1.15–6.62), p = 0.023] and the HbA1c level at the time of the OGTT in pregnancy [OR = 4.78 (1.19–19.20), p = 0.028] remained significant predictors for glucose intolerance postpartum. Women with glucose intolerance postpartum had a similar insulin sensitivity [Matsuda index 0.656 (0.386–1.224) vs. 0.778 (0.532–1.067), p = 0.709; 1/HOMA-IR 0.004 (0.002–0.009) vs. (0.004–0.003–0.007), p = 0.384] but a lower beta-cell function compared to women with a normal OGTT postpartum, remaining significant after adjustment for confounders [ISSI-2 1.6 (1.2–2.1) vs. 1.9 (1.7–2.4),p = 0.002].

**Conclusions:**

Glucose intolerance is very frequent in early postpartum in women with GDM based on the 2013 WHO criteria in a two-step screening strategy and these women have an impaired beta-cell function. Nearly one third of women did not attend the scheduled OGTT postpartum and these women have an adverse risk profile. More efforts are needed to engage and stimulate women with GDM to attend the postpartum OGTT.

## Introduction

Gestational diabetes (GDM) was historically defined as ‘any degree of glucose intolerance with onset or first recognition during pregnancy’ [1]. The incidence of GDM is rising globally and it represents an important modifiable risk factor for adverse pregnancy outcomes such as macrosomia and preeclampsia [2,3]. There is since long lack of uniformity in the approach to screening and diagnosis of GDM. Since 2010, the ‘International Association of Diabetes and Pregnancy Study Groups’ (IADPSG) recommends a one-step screening strategy with the 2-hour 75g oral glucose tolerance test (OGTT) using stricter criteria to diagnose GDM [4]. Using the IADPSG criteria, GDM affects 9–25% of pregnancies [5]. Since 2013 the World Health Organization (WHO) also advises the use of the IADPSG criteria for the diagnosis of GDM [6]. The IADPSG criteria for the diagnosis of GDM are therefore now commonly called the 2013 WHO criteria for GDM. In the past, research into whether women with GDM suffered from postpartum type 2 diabetes (T2DM) focused on women with GDM diagnosed by the 1999 WHO or the Carpenter and Coustan criteria. Using these criteria, GDM women were found to be seven times more likely to develop T2DM than non-GDM women [7]. The use of the 2013 WHO criteria for GDM results in a greater proportion of women diagnosed with GDM but there is uncertainty on the risk of women who have had mild GDM to develop T2DM postpartum. Timely detection of glucose intolerance postpartum is important since progression to T2DM can be reduced with 40% by lifestyle intervention programmes and metformin therapy post-pregnancy [8]. Currently there are few data on the risk for persistent glucose intolerance in early postpartum in women with GDM diagnosed by an universal two-step screening strategy and the 2013 WHO criteria. Studies are therefore needed that address this deficiency by including a postpartum OGTT in women diagnosed with GDM to establish early post-delivery (pre)diabetes status. The aim of our study was therefore to evaluate the uptake of our current screening strategy postpartum, the prevalence and the risk factors for glucose intolerance based on a OGTT three months postpartum in women with GDM detected by a universal two-step screening strategy with the 2013 WHO criteria.

## Materials and Methods

Retrospective analysis of the electronic medical records of the University Hospital UZ Leuven in Belgium, from 01-03-2014 till 08-02-2016 of women with a recent history of GDM diagnosed with the 2013 WHO criteria. The study was approved by the Institutional Review Board of UZ Leuven (S58329). The study was conducted according to the principles expressed in the Declaration of Helsinki. Due to the retrospective nature of the study there was no need for informed consent from the participants (the data were analyzed anonymously) as in compliance with the Belgian Law of December 8,1992 on the protection of privacy and the Belgian Law of August 22, 2002 on the rights of the patient.

Approximately 2400 women are delivered annually at our hospital. The background prevalence of T2DM in Belgium is 6.5% [9]. In the general adult population 28% of women are overweight and 13% are obese [10]. Leuven is a medium size city in the region of Flanders and has a population with a rather low background number of women from ethnic minorities (EM) (10.9%) [10]. Accurate data on the prevalence of GDM are lacking in Belgium and the current practice for screening for GDM varies across different centers [11]. Women are not yet universally screened for overt diabetes at first prenatal visit in our hospital.

### Screening for GDM

Until March 2014, a 100g 3-h OGTT with the Carpenter and Coustan criteria was used for the diagnosis of GDM in our hospital. Since March 2014, we use a 2-h 75g OGTT with the 2013 WHO criteria for the diagnosis of GDM. However, we continued to use a universal two-step screening strategy for GDM in our hospital with a non-fasting 50g glucose challenge test (GCT) (performed at the hospital or in primary care) between 24–28 weeks of pregnancy. Those testing positive [threshold after 1-h ≥ 140 mg/dl (7.8 mmol/l)] had a 2-h 75-g OGTT in the hospital at 24–28 gestational weeks using the 2013 WHO criteria for GDM [fasting plasma glycaemia (FPG) ≥ 92 mg/dl (5.1 mmol/l), 1-h glycaemia ≥ 180 mg/dl (10.0 mmol/l), 2-h glycaemia ≥ 153 mg/dl (8.5 mmol/l), diagnosis of GDM if ≥ 1 values is abnormal] [4]. There are no specific recommendations in our center on which women should receive screening for GDM before 24 weeks of pregnancy. Screening for GDM with a GCT is sometimes performed before 24 weeks of pregnancy in high risk women such as women with a history of GDM. As part of an ongoing study evaluating the one-step approach with a 75g OGTT using the 2013 WHO criteria, some women received the 75g OGTT irrespective of the GCT result [12]. Women with GDM were treated with insulin when despite lifestyle measures the FPG was ≥ 95 mg/dl (5.3 mmol/l) and/or 2-hour postprandial glycaemia ≥120 mg/dl (6.7 mmol/l). Oral anti-diabetes drugs such as metformin and glibenclamide (glyburide) are not routinely used during pregnancy in our hospital. Our primary goal was to evaluate the prevalence of glucose intolerance in early postpartum since the implementation of the 2013 WHO criteria for the diagnosis of GDM in our hospital. A formal power calculation was therefore not performed.

### Follow up postpartum

In our center women with a previous diagnosis of GDM are recommended to receive a 2 hour 75g OGTT three months after the delivery. Women receive an appointment for the postpartum OGTT at the delivery and a reminder is send with a SMS text message using an automatic electronic system within two weeks before the scheduled appointment. If women do not attend the scheduled postpartum OGTT, the diabetes nurse will try to reach women once more by telephone to make a new appointment. As part of the normal routine in our hospital, the OGTT is generally postponed to max. 6 months postpartum If women are still breastfeeding at 3 months postpartum.

### Registration of characteristics and pregnancy outcomes

Outcomes were obtained from review of the electronic database. Maternal characteristics recorded were age, weight, body mass index (BMI) at first prenatal visit and at delivery, overweight (BMI ≥25 Kg/m^2^), obesity (BMI ≥30 Kg/m^2^), weight gain (difference in weight between first prenatal visit and the delivery), ethnicity, parity, smoking and alcohol habits, family history of diabetes, history of GDM, history of polycystic ovary syndrome, hypertension, dyslipidemia and glucose intolerance before pregnancy. Excessive weight gain was defined according to the most recent Institute of Medicine (IOM) guidelines, except for obese women for whom we use the recommendation to gain ≤ 5Kg during pregnancy [13]. Other data that were recorded are: whether or not the scheduled postpartum OGTT was attended, the timing of the OGTT postpartum, the glucose values and the insulin values based on the 75g OGTT postpartum (0min-30min-60min-120min) and the glucose values based on the 75g OGTT during pregnancy (0min-60min-120min), whether women breastfed or not, gestational age at delivery, the timing and result of the GCT, the gestational age at the diagnosis of GDM, HbA1c at the time of the 75g OGTT during pregnancy, whether women received treatment with corticoids during pregnancy after the screening test, need of insulin, type of insulin and number of injections and the gestational age at the start of insulin. There are no data in our database on how long or how exclusive breastfeeding was given.

The following maternal pregnancy outcomes were recorded: gestational hypertension (blood pressure ≥ 140/90mmHg), preeclampsia [hypertension with proteinuria or in combination with reduced fetal growth or the ‘Hemolysis Elevated Liver enzymes and Low Platelets’ (HELPP)-syndrome], preterm delivery (<37 weeks of gestation) and cesarean section (planned + emergency sections combined). The following neonatal outcomes were recorded: birth weight, macrosomia (birth weight > 4kg), large-for-gestational age infants (LGA, birth weight >90 percentile adjusted for sex and parity according to the Flemish birth charts), small-for-gestational age infants (SGA, birth weight <10 percentile adjusted for sex and parity according to the Flemish birth charts), shoulder dystocia, Apgar score < 7 at five minutes and admission at the neonatal intensive care unit (NICU).

### Definition of (pre)diabetes

To define glucose intolerance postpartum, the criteria of the American Diabetes Association were used: diabetes was defined as FPG ≥126mg/dl (7.0mmol/l) and/or 2-hour glycaemia at the OGTT postpartum ≥200mg/dl (11.1mmol/l)] and prediabetes was defined as impaired fasting glucose (IFG) with a FPG ≥100-125mg/dl (5.5–6.9mmol/l) and/or impaired glucose tolerance (IGT) with a 2-hour glycaemia at the OGTT postpartum ≥140-199mg/dl (7.8–11.0mmol/l) [1].

### Insulin sensitivity and beta-cell function

Insulin sensitivity was measured using the insulin sensitivity index of Matsuda, a well-established measure of whole-body insulin sensitivity [14]. The insulin sensitivity index of Matsuda is defined as 10 000/√ [(FPG x fasting plasma insulin) X (mean glucose during OGTT x mean insulin during OGTT)] [15]. As a secondary measure of insulin sensitivity (largely hepatic), we also calculated the reciprocal of the homeostasis model assessment of insulin resistance (1/HOMA-IR) [15]. HOMA-IR is calculated as the product of FPG and fasting plasma insulin divided by 22.5 [15]. Beta-cell function was assessed by the insulinogenic index dived by HOMA-IR. The insulinogenic index was calculated as the incremental change in insulin concentration during the first 30 min of the OGTT divided by the incremental change in glucose during the same period [16,17]. As a secondary measure of beta-cell function, the insulin secretion sensitivity index (ISSI-2) was measured, an OGTT-derived measure that is analogous to the disposition index obtained from the frequently sampled intravenous glucose tolerance test [18,19]. Glycaemia was assessed by the area under the glucose curve during the OGTT, calculated using the trapezoidal rule [18,19]. ISSI-2 is defined as the product of 1) insulin secretion measured by the ratio of the area under the insulin curve to the area under the glucose curve and 2) insulin sensitivity measured by the insulin sensitivity index of Matsuda [18,19]. All these measures have been validated for use in women with GDM.

Until October 2015 HbA_1c_ was measured by a reversed-phase cation-exchange chromatography (ADAMS HA-8160, Menarini Diagnostics Benelux, Zaventem, Belgium). Since October 2015 HbA_1c_ was measured by a high performance liquid chromatography principle with the cationic non-porous ion exchanger using the ionic difference (Tosoh HLC-723G8, Tessendrlo, Belgium). In our center Hba1c is reported in compliance with the National Glycohemoglobin Standardization Program since 1996. Plasma glucose was measured by an automated colorimetric-enzymatic method (hexokinase-glucose-6-phosphate-dehydrogenase, application 668) on a Hitachi/Roche-Modular P analyzer. Insulin was measured by the immunometric ECLIA (Roche Modular E170, Basel, Switserland).

### Statistical Analyses

Statistical analyses were performed using SPSS 22.0. Continuous data were expressed as mean and standard deviation if normally distributed, non-parametric variables were expressed as median. Categorical data were expressed as percentage. To compare variables between two groups independent samples *t*-tests were used for normally distributed continuous variables, Mann–Whitney’s *U*-test for non-parametric variables and chi-squared tests for categorical variables. Multivariable logistic regression was used to analyse independent predictors for glucose intolerance postpartum. Due to a limited number of events, we used a max. number of 5 variables per multivariable regression model. We therefore analyzed the effect of each variable of interest in a separate model, including also age, BMI, ethnicity and breastfeeding as possible confounders. Multivariable logistic regression was also used to evaluate the impact of possible confounders on insulin sensitivity and beta-cell function. A p-value of <0.05 (two-tailed) was considered significant.

## Results

Over a period of 23 months, 211 women were identified with a recent history of GDM. After evaluation of the medical files, 20 files were not included in the analysis because women did not deliver in our center (10) or because they had not yet received the postpartum OGTT because they were not yet 3 months postpartum (10), leaving a cohort of 191 women with a recent history of GDM for analysis. Over this time period the prevalence of GDM was 5.5%. Overall, 49.5% of women had an FPG diagnostic for GDM using the 2013 WHO criteria.

The mean age of the cohort was 31.7 years (±4.8), 14.1% had a history of GDM, 9.9% had a history of hypertension, 6.3% had a history of prediabetes, 29.6% were overweight and 28.0% were obese at the first prenatal visit. Of all women 29.8% had a EM background. The most frequent EM background was Middle-Eastern in 11.5%, Black African in 6.8%, South Asian in 6.8% and Northern-African in 3.7%. The median week at diagnosis of GDM was 27.0 (25.0–28.0) and 23.2% of women needed insulin during pregnancy.

Mean gestational age at delivery was 38.1 ±2.2 weeks. Analysis of pregnancy outcomes showed that 6.3% had a LGA baby, 3.1% had macrosomia, 11.0% had a SGA baby, 13.6% had gestational hypertension, 4.7% had preeclampsia, 18.3% had a preterm delivery, 2.1% had shoulder dystocia, 4.2% had low Apgar scores, 15.2% was admitted at NICU and 34.0% was delivered by cesarean section.

Postpartum screening was offered to all women but 29.3% (56) did not attend the scheduled postpartum OGTT. Compared to women who received an OGTT postpartum, women who did not attend the postpartum OGTT, had a higher BMI (28.6 ±6.8 vs. 26.2 ± 5.6, p = 0.015), were more often from a EM background (41.1% vs. 25.2%, p = 0.029) and smoked more often during pregnancy (14.3% vs. 2.2%, p = 0.001) [Table 1].

**Table 1:** Comparison of the characteristics between women who received an OGTT postpartum and women who failed to attend the scheduled OGTT post-partum.

	Women with OGTT postpartum N = 135 (70.7%)	Women without OGTT postpartum N = 56 (29.3%)	p-value
Age (mean)	31.5±4.6	32.2±5.8	0.370
BMI at first prenatal visit (mean)	26.2±5.6	28.6±6.8	**0.015**
% overweight at first prenatal visit	28.6%	32.1%	0.623
% obese at first prenatal visit	24.8%	35.7%	0.128
% excessive weight gain	33.8%	48.2%	0.063
% EM	25.2%	41.1%	**0.029**
% smoking before pregnancy	6.7%	17.9%	**0.019**
% smoking during pregnancy	2.2%	14.3%	**0.001**
% first degree family member with T2DM	11.9%	19.6%	0.362
% history of GDM	13.3%	16.1%	0.257
% hypertension before pregnancy	10.4%	8.9%	0.762
% prediabetes before pregnancy	5.2%	8.9%	0.181
% multiparous	60.0%	67.9%	0.308
% breastfeeding	77.8%	78.6%	0.904
Week OGTT	27.0(26.0–28.0)	26.5(25.0–28.0)	0.515
% fasting abnormal on the OGTT in pregnancy	45.0%	60.4%	0.059
% ≥2 values abnormal on the OGTT in pregnancy	40.3%	48.1%	0.328
% ≥3 values abnormal in pregnancy	9.3%	18.5%	0.080
% insulin	23.1%	23.2%	0.990
Weeks start insulin (median)	29.0(27.0–30.5)	28.0(20.0–30.5)	0.494

GDM: gestational diabetes; OGTT: oral glucose tolerance test; EM: Ethnic Minority Backgrounds; T2DM: type 2 diabetes; Statistical analyzes: for normally distributed continuous variables the independent samples *t*-tests were used and chi-squared tests were used for categorical variables. P values in bold are statistical significant.

The postpartum OGTT was performed at a median of 14.0 weeks (13.0–15.0). Of all women (135) who received an OGTT postpartum, 42.2% (57) had prediabetes (11.9% IFG, 24.4% IGT and 5.9% both IFG and IGT combined) and 1.5% (2) had overt diabetes. Compared to women with a normal OGTT postpartum, women with glucose intolerance were older (32.5±4.3 vs. 30.8±4.8 years, p = 0.049), were more often obese (34.5% vs. 17.3%, p = 0.023), had more often excessive weight gain during pregnancy (44.8% vs. 25.3%, p = 0.018), were more often from an EM background (33.9% vs. 18.4%, p = 0.040), less often breastfed (69.5% vs. 84.2%, p = 0.041) and had more often an abnormal fasting glycaemia (55.6% vs. 37.3%, p = 0.040) and higher median HbA1c [(5.1% (5.0–5.4) vs. 5.0%(4.8–5.2), p = 0.001] at the time of the OGTT [Table 2]. In the multivariable logistic regression, an EM background [OR = 2.76 (1.15–6.62), p = 0.023] and the HbA1c level at the time of the OGTT in pregnancy [OR = 4.78 (1.19–19.20), p = 0.028] remained significant independent predictors for glucose intolerance postpartum after adjustment for BMI, ethnicity, age and breastfeeding [Table 3].

**Table 2:** Comparison of the characteristics between women with a normal OGTT and women with glucose intolerance postpartum.

	Normal N = 76 (56.3%)	Glucose intolerance N = 59 (43.7%)	p-value
Age (mean)	30.8±4.8	32.5±4.3	**0.049**
BMI at first prenatal visit (mean)	25.4±5.5	27.2±5.6	0.069
% overweight at first prenatal visit	30.7%	25.9%	0.543
% obese at first prenatal visit	17.3%	34.5%	**0.023**
% excessive weight gain	25.3%	44.8%	**0.018**
Weight gain in Kg (mean)	15.0± 1.7	15.4±2.0	**0.658**
% EM	18.4%	33.9%	**0.040**
% smoking before pregnancy	5.3%	8.5%	0.458
% first degree family member with T2DM	11.8%	11.9%	0.984
% history of GDM	13.2%	13.6%	0.946
% history of hypertension	6.6%	15.3%	0.101
% history of prediabetes	3.9%	6.8%	0.462
% multiparous	60.5%	59.3%	0.887
% breastfeeding	84.2%	69.5%	**0.041**
Week of GCT (median)	25.5(24.2–26.2)	25.0(24.0–26.7)	0.671
Result GCT mg/dl (median)	153.0(144.0–174.0)	169.0(153.5–187.5)	0.059
Week OGTT (median)	153.0 (144.0–174.0)	26.2(25.0–27.6)	0.140
% fasting abnormal on the OGTT in pregnancy	37.3%	55.6%	**0.040**
% 1 hour abnormal on the OGTT in pregnancy	48.0%	40.7%	0.414
% 2 hour abnormal on the OGTT in pregnancy	62.7%	53.7%	0.307
% ≥2 values abnormal on the OGTT in pregnancy	40.0%	40.7%	0.933
% ≥3 values abnormal on the OGTT in pregnancy	9.3%	9.3%	0.989
HbA1c % [mmol/mol] at time of OGTT (median)	5.0%(4.8–5.2) [31(29–33)]	5.1%(5.0–5.4) [32(31–36)]	**0.001**
% insulin	18.7%	28.8%	0.167
Weeks start insulin (median)	29.2(27.7–31.0)	28.0(25.0–30.2)	0.316

GDM: gestational diabetes; OGTT: oral glucose tolerance test; GCT: glucose challenge test; EM: Ethnic Minority Backgrounds; T2DM: type 2 diabetes; Statistical analyzes: for normally distributed continuous variables the independent samples *t*-tests were used, for non-parametric variables the Mann–Whitney’s *U*-test and for categorical variables the chi-squared tests. P values in bold are statistical significant.

**Table 3:** Independent predictors of glucose intolerance postpartum.

Variable	Degrees of freedom	Standard Error	OR (95% CI)	p-value
age	1	0.43	1.07(0.98–1.16)	0.123
BMI	1	0.036	1.04(0.97–1.12)	0.218
EM	1	0.45	2.76(1.15–6.62)	**0.023**
breastfeeding	1	0.47	0.44 (0.17–1.11)	0.082
excessive gestational weight gain	1	0.44	2.26(0.96–5.35)	0.062
Smoking	1	0.79	1.39(0.26–6.59)	0.677
first degree family member with T2DM	1	0.61	0.91(0.28–2.99)	0.877
history of GDM	1	0.59	0.74(0.23–2.4)	0.739
history of hypertension	1	0.65	2.35(0.66–8.42)	0.188
history of prediabetes	1	0.87	0.97(0.18–5.31)	0.971
multiparity	1	0.44	0.53(0.22–1.25)	0.149
Week of GCT	1	0.20	0.85(0.57–1.27)	0.426
Result GCT	1	0.01	1.01(0.98–1.03)	0.525
Week OGTT	1	0.06	0.94(0.83–1.06)	0.287
Fasting glycaemia on OGTT in pregnancy	1	0.02	1.03(0.98–1.07)	0.240
1h glycaemia on OGTT in pregnancy	1	0.01	1.00(0.99–1.01)	0.559
2h glycaemia on OGTT in pregnancy	1	0.01	0.99(0.98–1.00)	0.125
≥2 values abnormal on the OGTT in pregnancy	1	0.40	0.71(0.32–1.57)	0.397
≥3 values abnormal on the OGTT in pregnancy	1	0.68	0.64(0.17–2.44)	0.518
HbA1c at time of OGTT in pregnancy	1	0.71	4.78(1.19–19.20)	**0.028**
insulin	1	0.47	1.16(0.46–2.93	0.753
Weeks start insulin	1	0.11	0.97(0.78–1.21)	0.801

GDM: gestational diabetes; OGTT: oral glucose tolerance test; GCT: glucose challenge test; EM: Ethnic Minority Backgrounds; T2DM: type 2 diabetes; Multivariable logistic regression with odds ratio (OR) adjusted for age, BMI, ethnicity and breastfeeding. Data are presented as OR (95% CI lower-upper limit). P values in bold are statistical significant.

Compared to women with a normal OGTT postpartum, women with glucose intolerance postpartum had a similar insulin sensitivity but a lower beta-cell function, remaining significant after adjustment for confounders [ISSI-2 1.6 (1.2–2.1) vs. 1.9 (1.7–2.4),p = 0.002] [Table 4].

**Table 4:** Comparison of the beta-cell function and insulin sensitivity between women with a normal OGTT and women with glucose intolerance postpartum.

	Normal N = 76 (56.3%)	Glucose intolerance N = 59 (43.7%)	P value	Adjusted p value
ISSI-2 index (median)	1.9(1.7–2.4)	1.6(1.2–2.1)	**<0.0001**	**0.002**
Insulinogenic index/HOMA-IR index (median)	0.010(0.008–0.014)	0.009(0.005–0.015)	0.119	0.249
Matsuda index (median)	0.778(0.532–1.067)	0.656(0.386–1.224)	0.279	0.709
1/HOMA-IR index(median)	0.004(0.003–0.007)	0.004(0.002–0.009)	0.699	0.384

ISSI-2: insulin secretion sensitivity index postpartum; Insulinogenic index/HOMA-IR is a measure for beta-cell function postpartum; Matsuda: insulin sensitivity index of Matsuda postpartum; 1/HOMA-IR: the reciprocal of the homeostasis model assessment of insulin resistance post-partum; Statistical analyzes: for non-parametric variables the Mann–Whitney’s *U*-test were used. The p-values for the measurements post-partum are adjusted for age, BMI, ethnicity and breastfeeding. P values in bold are statistical significant.

Compared to women with IGT postpartum, women with IFG, were more often obese (62.6% vs. 21.9%, p = 0.006), had less often a history of GDM (14.1% vs. 21.2%, p = 0.047), had a lower GCT value and a higher fasting but lower 2 hour value at the OGTT in pregnancy [Table 5]. There were no differences between both groups in insulin sensitivity and beta-cell function [Table 6].

**Table 5:** Comparison of characteristics between women with an impaired fasting glucose (IFG) and women with an impaired glucose tolerance (IGT) postpartum.

	IFG N = 16 (32.7%)	IGT N = 33 (67.3%)	p-value
Age (mean)	32.0±5.3	33.5±3.6	0.255
BMI at first prenatal visit (median)	31.3±4.4	25.2±4.8	**<0.0001**
% overweight at first prenatal visit	37.5%	21.9%	0.251
% obese at first prenatal visit	62.6%	21.9%	**0.006**
% excessive weight gain	68.8%	34.4%	**0.024**
% EM	25.0%	39.4%	0.321
% first degree family member with T2DM	18.8%	12.1%	0.659
% history of GDM	14.1%	21.2%	**0.047**
% hypertension before pregnancy	12.5%	9.1%	0.712
% prediabetes before pregnancy	0	9.1%	0.213
% multiparous	50.0%	60.6%	0.482
% breastfeeding	68.8%	69.7%	0.946
% progestin-only oral contraceptive	69.2%	44.4%	0.380
Week of GCT (median)	25.5(24.6–27.1)	25.0(24.0–27.5)	0.667
Result GCT (median)	147.0(120.7–163.5)	170.0(156.0–192.0)	**0.016**
Week OGTT (median)	27.0(24.0–30.5)	26.0(25.0–27.1)	0.177
% fasting abnormal on the OGTT in pregnancy	80.0%	43.3%	**0.020**
% 1h abnormal on the OGTT in pregnancy	40.0%	40.0%	1.000
% 2h abnormal on the OGTT in pregnancy	33.3%	66.7%	**0.034**
% ≥2 values abnormal on the OGTT in pregnancy al	40.0%	46.7%	0.671
% ≥3 values abnormal on the OGTT in pregnancy	13.3%	3.3%	0.205
Median FPG on the OGTT in pregnancy	95.0(93.0–101.0)	90.5(81.0–92.0)	**0.012**
Median 1 hour glycaemia on the OGTT in pregnancy	150.0(138.0–205.0)	172.0(163.7–196.0)	0.306
Median 2 hour glycaemia on the OGTT in pregnancy	115.0(110.0–158.0)	158.0(144.2–166.2)	**0.006**
% insulin	31.3%	21.2%	0.444
Weeks start insulin (median)	28.0(20.5–32.5)	29.0(27.0–31.0)	0.530

Eight women with the combination of IFG and IGT were excluded from these analyses. GDM: Gestational diabetes; OGTT: oral glucose tolerance test; GCT: glucose challenge test; FPG: fasting plasma glycaemia; EM: Ethnic Minority Backgrounds; T2DM: type 2 diabetes; Statistical analyzes: for normally distributed continuous variables the independent samples *t*-tests were used, for non-parametric variables the Mann–Whitney’s *U*-test and for categorical variables the chi-squared tests. P values in bold are statistical significant.

**Table 6:** Comparison of the beta-cell function and the insulin sensitivity between women with an impaired fasting glucose (IFG) and women with an impaired glucose tolerance (IGT) postpartum.

	IFG N = 16 (32.7%)	IGT N = 33 (67.3%)	P value	Adjusted p value
**ISSI-2 index (median)**	1.7(1.1–2.2)	1.6(1.3–2.1)	0.890	0.610
**Insulinogenic index/HOMA-IR index (median)**	0.007 (0.004–0.015)	0.011 (0.006–0.017)	0.363	0.831
**Matsuda index (median)**	0.441 (0.356–1.527)	0.696 (0.414–1.160)	0.396	0.977
**1/HOMA-IR (median)**	0.003 (0.002–0.007)	0.005 (0.002–0.009)	0.209	0.737

Eight women with the combination of IFG and IGT were excluded from these analyses. IFG: impaired fasting glucose; IGT: impaired glucose tolerance; ISSI-2: insulin secretion sensitivity index postpartum; Insulinogenic index/HOMA-IR is a measure for beta-cell function postpartum; Matsuda: insulin sensitivity index of Matsuda postpartum; 1/HOMA-IR: the reciprocal of the homeostasis model assessment of insulin resistance post-partum; Statistical analyzes: for non-parametric variables the Mann–Whitney’s *U*-test were used. The p-values for the measurements post-partum are adjusted for age, BMI, ethnicity and breastfeeding. P values in bold are statistical significant.

## Discussion

There is uncertainty about how detection of GDM using the 2013 WHO criteria influences the longer term risk of developing T2DM in women. The use of the 2013 WHO criteria for GDM results in a greater proportion of women diagnosed with mild forms of GDM which might presumably lead to a lower proportion at risk for postpartum glucose intolerance. Previous studies have evaluated the risk of postpartum glucose intolerance in women with a diagnosis of GDM based on a universal one-step approach with a 75g OGTT and the 2013 WHO criteria [20,21]. The use of the 2013 WHO criteria for GDM in a two-step screening strategy with a GCT is used as a practical solution so that the number of OGTTs can be limited but this has not yet been validated. Data on the sensitivity and specificity of a GCT in combination with a 75g OGTT and the 2013 WHO criteria are lacking. Ongoing studies such as the Belgian Diabetes in Pregnancy study (BEDIP-N) are currently evaluating the 2013 WHO criteria in a multiethnic Belgian cohort comparing a universal one-step, a two-step with a GCT and a risk factor based screening approach [12]. Data on the risk of glucose intolerance in early postpartum in women with GDM diagnosed with the two-step screening strategy and the 2013 WHO criteria are therefore needed. We show now similar high rates of glucose intolerance with the 2013 WHO criteria as with the Carpenter and Coustan criteria in early postpartum in our population [22]. In our cohort with GDM based on the 2013 WHO criteria, 42.2% had prediabetes (11.9% IFG, 24.4% IGT and 5.9% both IFG and IGT combined) and 1.5% had overt diabetes in early postpartum. We have previously reported that when using the 100g OGTT and the Carpenter and Coustan criteria for GDM in our population, 39.1% had prediabetes and 5.3% had diabetes in early postpartum [23]. The high prevalence of glucose intolerance in early postpartum using the Carpenter and Coustan criteria for GDM is to be expected since these criteria were developed to identify women at high risk for the development of T2DM after the delivery [22]. On the other hand, the IADPSG/2013 WHO criteria are the first diagnostic criteria for GDM based on the risk for adverse pregnancy outcomes. Adverse pregnancy outcomes in our study were low with LGA rates of 6.3% and macrosomia rates of 3.1%. Recent analysis of pregnancy outcomes in our center in women with GDM diagnosed with the Carpenter and Coustan criteria showed much higher LGA and macrosomia rates (resp. 16.9% and 8.7%) [24]. This is in line with previous studies showing lower adverse pregnancy outcomes in women treated for GDM based on the 2013 WHO criteria compared to the Carpenter and Coustan criteria [25]. In our study 23.2% of women needed treatment with insulin during pregnancy. This is in line with a large Spanish cohort study showing similar rates of insulin use (around 20%) in women with GDM based on the 2013 WHO criteria [25].

Our study shows much higher rates of glucose intolerance in early postpartum compared to studies in Irish women where a rate of prediabetes and diabetes of 19% was reported in women with GDM based on the 2013 WHO criteria in the first year postpartum and this increased to 25% in the same cohort when re-screened up to 5 years post-delivery [20,21]. This may be related to differences in population characteristics since only 13.6% of the Irish women had an EM background while this was 29.8% in our study. The main difference is probably related to the screening strategy used for GDM. In the Irish studies a universal one-step approach with a 75g OGTT was used while in our center a universal two-step screening strategy with a GCT was used which might identify women at higher risk for the development of glucose intolerance postpartum. Previous studies have shown that any degree of abnormal glucose homeostasis in pregnancy independently predicts an increased risk of glucose intolerance and that women with GDM, with gestational impaired glucose tolerance or with an abnormal GCT but normal OGTT have varying rates of declining beta-cell function with a progressive decline of beta-cell function over time across the different groups [26,27]. GDM women diagnosed in a two-step screening strategy fail both the GCT and OGTT test and this might therefore identify women with a lower underlying beta-cell function compared to women with mild GDM detected by OGTT only. Our study shows that compared to women with a normal OGTT, women with glucose intolerance had a similar insulin sensitivity but a lower beta-cell function postpartum, which remained significantly lower after adjustment of confounders such as age, BMI, ethnicity and breastfeeding.

Of all women with prediabetes, 57.9% had IGT, 28.1% had IFG and 14.0% had IFG and IGT combined. A FPG alone postpartum would therefore have missed the majority of women with glucose intolerance, confirming the need for an OGTT in early postpartum in our population. This is in contrast with the most recent British guidelines of the ‘National Institute for Health and Care Excellence’ (NICE) stating that a 75g OGTT should not be routinely offered for women with a history of GDM and instead recommending the use of a fasting glucose in early postpartum [28]. Studies evaluating the use of HbA1c alone or in combination with FPG to diagnose glucose intolerance in women who have had GDM, show conflicting results with sensitivity rates of HbA1c and FPG combined ranging from 83.0% to 90.0% [21,29].

The most important risk factors to develop glucose intolerance in early postpartum differ according to the populations studied [30]. The most common risk factors are maternal age, pre-pregnancy weight, early GDM diagnosis, insulin treatment during pregnancy and the FPG on the diagnostic OGTT during pregnancy [30]. In our study, only an EM background and the HbA1c level at the time of diagnosis of GDM remained independent predictors of glucose intolerance after adjustment for confounders. Breastfeeding has been associated with lower FPG and insulin, and a lower prevalence of glucose intolerance 6–9 weeks post-partum [21,31]. The majority of women were breastfeeding in our cohort. Compared to women with a normal OGTT postpartum, fewer women were breastfeeding in the group with glucose intolerance. However, in the multivariable regression analysis, breastfeeding was not an independent predictor for glucose intolerance. This might be due to the lack of data in our database on how long or how exclusive women were effectively breastfeeding postpartum.

We also show that compared to women with IGT postpartum, women with IFG are more often obese and have a higher FPG at the time of the OGTT in pregnancy. We have previously shown that after a diagnosis of GDM with the Carpenter and Coustan criteria women with IFG in early postpartum, have a lower insulin sensitivity compared to women with IGT and this seems to be largely driven by a higher BMI [23]. In current study, there were no differences between both groups in insulin sensitivity and beta-cell function. This might be related to the smaller sample size and the use of the 2013 WHO criteria for GDM diagnosing more milder forms of GDM.

In our study nearly one third of women did not attend the scheduled OGTT postpartum. This is higher than the non-attendance rate of 21% we have previously reported in women with GDM based on the Carpenter and Coustan criteria [23]. More efforts are clearly needed to better inform and engage women to attend the postpartum test since despite the use of a an automatic electronic system that triggers postpartum SMS reminders and if needed a telephonic recall done by a diabetes nurse, the attendance rate seems to decrease. Especially women with an EM background have a high non-attendance rate in our study while we show that these women are at particular high risk for glucose intolerance in early postpartum. Many studies have reported even lower postpartum testing rates with only 30–50% of women with recent GDM who receive an OGTT within 6 months after the delivery [32,33]. This is a missed opportunity in a high-risk population to timely detect glucose intolerance and start lifestyle interventions to prevent or delay the development of glucose intolerance and diabetes [8].

Strengths of the study are the detailed characterization of a relatively large cohort of women with recent GDM based on a two-step screening strategy and the 2013 WHO criteria using a good database. A multivariable regression analysis was used to evaluate independent predictors of glucose intolerance postpartum. Multiple measures of beta-cell function and insulin sensitivity were calculated and adjusted for confounders. A limit of the study is the retrospective nature of the analysis and the lack of longer term data postpartum on the risk for glucose intolerance. We have no data on the HbA1c level postpartum and on weight loss of women postpartum, both possible predictors of glucose intolerance.

In conclusion, we show that glucose intolerance is very frequent in early postpartum in women with GDM based on the 2013 WHO criteria in a two-step screening strategy and these women have an impaired beta-cell function. Nearly one third of women did not attend the scheduled OGTT postpartum. More efforts are needed to engage and stimulate women with GDM to attend the postpartum OGTT.
